# Biometric handwriting analysis to support Parkinson’s Disease assessment and grading

**DOI:** 10.1186/s12911-019-0989-3

**Published:** 2019-12-12

**Authors:** Giacomo Donato Cascarano, Claudio Loconsole, Antonio Brunetti, Antonio Lattarulo, Domenico Buongiorno, Giacomo Losavio, Eugenio Di Sciascio, Vitoantonio Bevilacqua

**Affiliations:** 1 Department of Electrical and Information Engineering (DEI), Polytechnic University of Bari, Italy, Via Edoardo Orabona, 4, Bari, Italy; 2 Apulian Bioengineering s.r.l., Via delle Violette 14, Modugno (BA), Italy; 3 Medica Sud s.r.l., Via della Resistenza, 82, Bari, Italy

**Keywords:** Handwriting analysis, Model-free, SEMG, Parkinson disease, ANN, MOGA

## Abstract

**Background:**

Handwriting represents one of the major symptom in Parkinson’s Disease (PD) patients. The computer-aided analysis of the handwriting allows for the identification of promising patterns that might be useful in PD detection and rating. In this study, we propose an innovative set of features extracted by geometrical, dynamical and muscle activation signals acquired during handwriting tasks, and evaluate the contribution of such features in detecting and rating PD by means of artificial neural networks.

**Methods:**

Eleven healthy subjects and twenty-one PD patients were enrolled in this study. Each involved subject was asked to write three different patterns on a graphic tablet while wearing the Myo Armband used to collect the muscle activation signals of the main forearm muscles. We have then extracted several features related to the written pattern, the movement of the pen and the pressure exerted with the pen and the muscle activations. The computed features have been used to classify healthy subjects versus PD patients and to discriminate mild PD patients from moderate PD patients by using an artificial neural network (ANN).

**Results:**

After the training and evaluation of different ANN topologies, the obtained results showed that the proposed features have high relevance in PD detection and rating. In particular, we found that our approach both detect and rate (mild and moderate PD) with a classification accuracy higher than 90%.

**Conclusions:**

In this paper we have investigated the representativeness of a set of proposed features related to handwriting tasks in PD detection and rating. In particular, we used an ANN to classify healthy subjects and PD patients (PD detection), and to classify mild and moderate PD patients (PD rating). The implemented and tested methods showed promising results proven by the high level of accuracy, sensitivity and specificity. Such results suggest the usability of the proposed setup in clinical settings to support the medical decision about Parkinson’s Disease.

## Background

Parkinson’s Disease (PD) is the second most common neurodegenerative disorder after Alzheimer’s disease that leads to several neuro-motor deficits. It is well known that PD patients exhibit problems when the perform movements that are executed sequentially due to the loss of coordination among the motor sequence components [1–5]. As a result, sequential movements are more segmented and characterized by pauses between sub-movements [6].

The handwriting is a task composed of sequential movements that involves fine and complex manual skills relying on a sophisticated mix of cognitive, sensory and motor components [7]. This explains the manifestation of abnormal features in the handwriting of PD patients. The difficulties in the handwriting process affecting PD patients are mainly two:

- difficulties related to the control of the movement amplitude, e.g. decreasing the size of the characters (micrographia) and failing in keeping the stroke width of the characters constant as the writing progresses [8–15];
- not regular and bradykinetic movements that lead to an increased movement duration, decreased speed and accelerations, and unstable velocity and acceleration [16–21].

Several research groups have investigated the use of handwriting’s features to classify PD patients and healthy subjects.

Helsper et al. published a study that investigated the handwriting differences between preclinical PD patients and healthy controls [22]. The authors analysed two lines of the handwritten text (sampled from a longer written text) and proposed an approach that considers (1) the extraction of 10 features from text segments written by test subjects as a first step, and then (2) the computation of a single resulting feature set based on the mean, the standard deviation and the frequency of the occurrences. The authors statistically proved the existence of features characterizing many years before the diagnosis.

Longstaff and collegues studied the relation between the inclination of PD patients to scale the character size and reduce the speed of drawing movements and the movement variability [23]. The experiment is based on the analysis of several geometrical writing patterns with different shape and size drawn with a pen on a graphics tablet. By analysing the extracted features the authors stated that there is a substantial divergence in the quality of movements between PD patients and healthy people

A different recording set-up has been used by Ünlü et al. [24] that recorded the pressure and the inclination of an electronic pen during writing tasks. Their proposed approach considered the extraction of 8 different features and the use the Receiver Operating Characteristic (ROC) to analyse the diagnostic possibilities both in term of sensitivity and specificity. Their results showed that the most representative feature is based on the difference between the writing pressure and the tremor of the pen tilt angle.

Electronic pen and tablet have been also used by Rosenblum et al. to collect the position, the pressure and the angle of the pen tip during the writing of two main patterns (i.e. the name and fixed address) [25]. The average values of the pressure and velocity acquired during the entire task and other spatial and temporal characteristics of each stroke allowed them to differentiate PD patients from control subjects with a sensitivity of 95.0%.

In our preliminary previous works [26, 27], we proposed a promising method to classify PD patients from healthy subjects by using only 4 features extracted by scanned text (through image processing techniques) and surface ElectroMyoGraphy (sEMG) signals. Recently, we used a graphic tablet and the Myo armband to extract biometric signals related to pen movements (pen tip position, inclination and pressure) and muscle activation [28, 29]. These signals were processed to extract a number of features used as input to two different classifiers.

In this work, we improve our recent studies by proposing a larger feature set and testing our classifiers on bigger cohort of subjects. Furthermore, we focused on the selection of the most representative features that better highlight the handwriting differences between (1) mild and moderate PD patients, and (2) PD patients and healthy subjects.

## Methods

The proposed model-free technique for the analysis of handwriting is computer-assisted and based on the extraction of features from biometrical signals [30] (i.e., sEMG signals, pen tilt, etc.) related to hand movements during handwriting tasks.

In the following sections, the features selected and used in our technique and the algorithms used for feature selection and classification are presented and described.

### Handwriting feature extraction

**Fig. 1:**
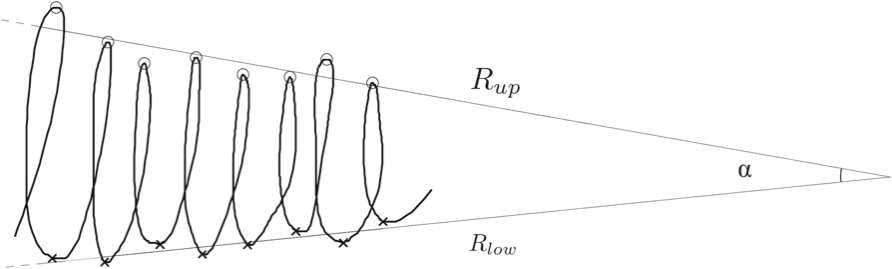
Representation of the regression lines *R*_*up*_ and *R*_*low*_ and the angle *α*. Circle and cross marks identifies respectively upper and lower peaks of the Y-coordinate of the pen tip position
    2 $$\begin{array}{@{}rcl@{}}  \overline{y}&=&\frac{1}{n}\sum^{n}_{i}y_{i}, \\ SST&=&\sum_{i}(y_{i}-\overline{y})^{2}, \\ SSE&=&\sum_{i}(y_{i}-\widehat{y_{l}})^{2}, \\ R^{2}&=&1-\frac{SSE}{SST}. \end{array} $$
    The three resulting patterns chosen as descriptors of the variability of the writing size are the *α* angle between the regression lines and the two coefficients of determination (*R*^2^).
    For the spiral patterns, instead, the feature is based on the variability of the strokes. We computed the vector $\overrightarrow {r}$ originating in *P* with respect to the spiral centroid point *C* for each point P of the X-Y pen tip position. Then, we calculated the angle *β* between $\overrightarrow {r}$ and the direction vector $\overrightarrow {d}$ tangent to the spiral in *P*. The visual explanation of the process is depicted in Fig. 2. Lastly, we calculated the standard deviation of the *β* angles computed for each point *P*. The standard deviation value is the feature chosen to describe the precision of the spiral.

Handwriting features have been derived from biometric signals obtained during handwriting tasks. In general, the proposed features can be grouped into two groups based on sEMG and pen tip signals:

- *Features derived from sEMG signals* – these features are related to the subject’s muscle activity and are derived from the sEMG signals obtained from the forearm of the subject:
  - *RMS features*: for every sEMG channel, Root Mean Square (RMS) is computed. It is determined by Eq. (1), where *x*_*i*_ is the sample value at the discrete-time *i*, and *n* is the total number of samples acquired.
    1 $$  RMS=\sqrt{\frac{1}{n}\sum^{n}_{i=1}x^{2}_{i}}  $$
  - *ZC features*: Zero Crossing (ZC) is a variance-related index. In detail, it is the number of sign variations between two consecutive samples, and it is only increased if the difference exceeds a predefined tolerance value. Considering two consecutive samples *x*_*k*_ and *x*_*k*+1_, the ZC value is increased if and only if the following condition is satisfied: *x*_*k*_>0 and *x*_*k*+1_<0, or *x*_*k*_<0 and *x*_*k*+1_>0 and |*x*_*k*_−*x*_*k*+1_|≥*tol*. Due to the presence of noisy signals, the tolerance value (*tol*) is used to prevent ZC increment; it is measured as the average of the standard deviations of all the sEMG channels considered. In addition, its value is divided by the length of the signal to normalize the features among the subjects.
- *Pen tip related features* - these features are extracted during the handwriting task from the signals produced by a graphic tablet:
  - *Cartesian and XY features*: these features refer to the pen tip writing kinematics on the graphic tablet and are derived from the orientation of the XY axes. This group of features includes the features extracted from the following signals: Cartesian and XY-velocity, Cartesian and XY-acceleration, Cartesian and XY-jerk. The features are determined as first, second and third derivatives, respectively, starting from the X-Y position. It leads to nine output signals.
  - *Pen tip pressure feature*: this is a scalar feature which refers to the pressure applied to the tablet surface by the pen tip.
  - *Azimuth and altitude feature*: the azimuth feature is the angle value between a reference direction (e.g., the Y axes of the tablet) and the pen direction projected on the horizontal plane. The altitude feature is the angle value between the pen direction and the horizontal plane.
  - *Pattern specific features*: these features are related to the writing size of letter-based patterns and the writing precision of spiral writing ones. In detail, for the letter-based patterns, the pen tip Y coordinate has been processed, and the upper and lower peaks are then determined. The two groups of peak points are then independently used to determine the upper regression line *R*_*up*_ and the lower regression line *R*_*low*_ using linear regression. Finally, the *α* angle between the regression lines is computed. The graphical representation of the procedure is shown in Fig. 1. A further feature taken into account is the coefficient of determination (*R*^2^) computed according to Eqs. (2).

**Fig. 2:**
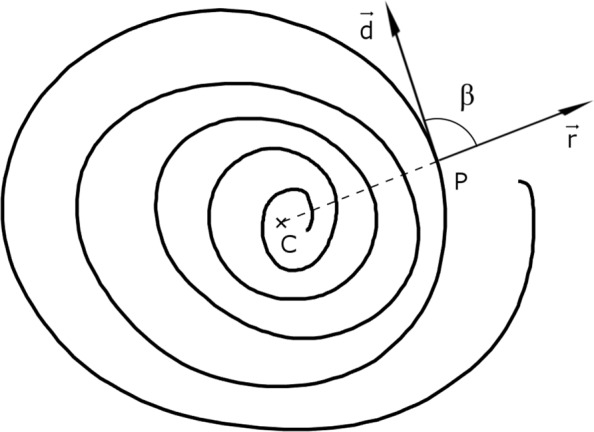
Example of computation of the spiral precision index *β*

### Feature selection and classification

A feature selection procedure has been used to understand the main representative for the subject’s status [31]; we used an approach similar to the entropy criterion (i.e., information gain) and based on a classification decision tree technique with Gini’s diversity index [32].

The classification procedure is based on Artificial Neural Network (ANN) classifier [33–35]. Since designing the topology of neural classifiers is challenging, several works have been proposed dealing with this task [36–38]. In this work, the topology of the classifier was optimised by the evolutionary approach proposed in [39].

We used a Multi-Objective Genetic Algorithm (MOGA) to find the optimal network topology. A genetic algorithm is a powerful optimization technique that reflects the process of natural selection where the fittest individuals are selected for reproduction in order to produce offspring of the next generation, thus explaining its feasibility in several research domains [40, 41]. The MOGA algorithm was configured to find the optimum varying the following network parameters: (i) number of hidden layers (integer interval: 1 to 3), (ii) number of neurons per layer (integer interval: 1 to 256 for the first hidden layer and 0 to 255 for other ones), and (iii) activation functions for each layer (one among: log-sigmoid - logsig, hyperbolic tangent sigmoid -tansig, pure linear - purelin and symmetric saturating linear - satlins).

The overall performances of MOGA algorithm and classification network were evaluated using the confusion matrix reported in Table 1 and the performance indexes reported in Eqs. 3, 4 and 5.

3 $$\begin{array}{@{}rcl@{}}  Accuracy&=&\frac{TP+TN}{TP+TN+FP+FN}, \end{array} $$

**Table 1:** The configuration of confusion matrix

	Positive	Negative
Positive	TP	FP
Negative	FN	TN

4 $$\begin{array}{@{}rcl@{}}  Specificity&=&\frac{TN}{TN+FP}, \end{array} $$

5 $$\begin{array}{@{}rcl@{}}  Sensitivity&=&\frac{TP}{TP+FN}. \end{array} $$

## Experiments and results

### Participants

32 participants (21 males, 11 females, age: 71.4 ±8.3 years old) were enrolled for the experiment. In detail, the participants were composed of 21 PD subjects (17 males and 4 females, age: 72.1 ±8.3) and 11 healthy ones (4 males and 7 females, age: 70.2 ±10.2 years old); the healthy group was selected to match the age of the PD one. The PD group was subsequently divided into mild and moderate subgroups according to the degree of the disease. The subgroups were composed of 12 mild patients (9 males and 3 females, age: 70.5 ±10.0) and 9 moderate ones (8 males and 1 female, age: 73.8 ±6.0).

### System set-up

**Fig. 3:**
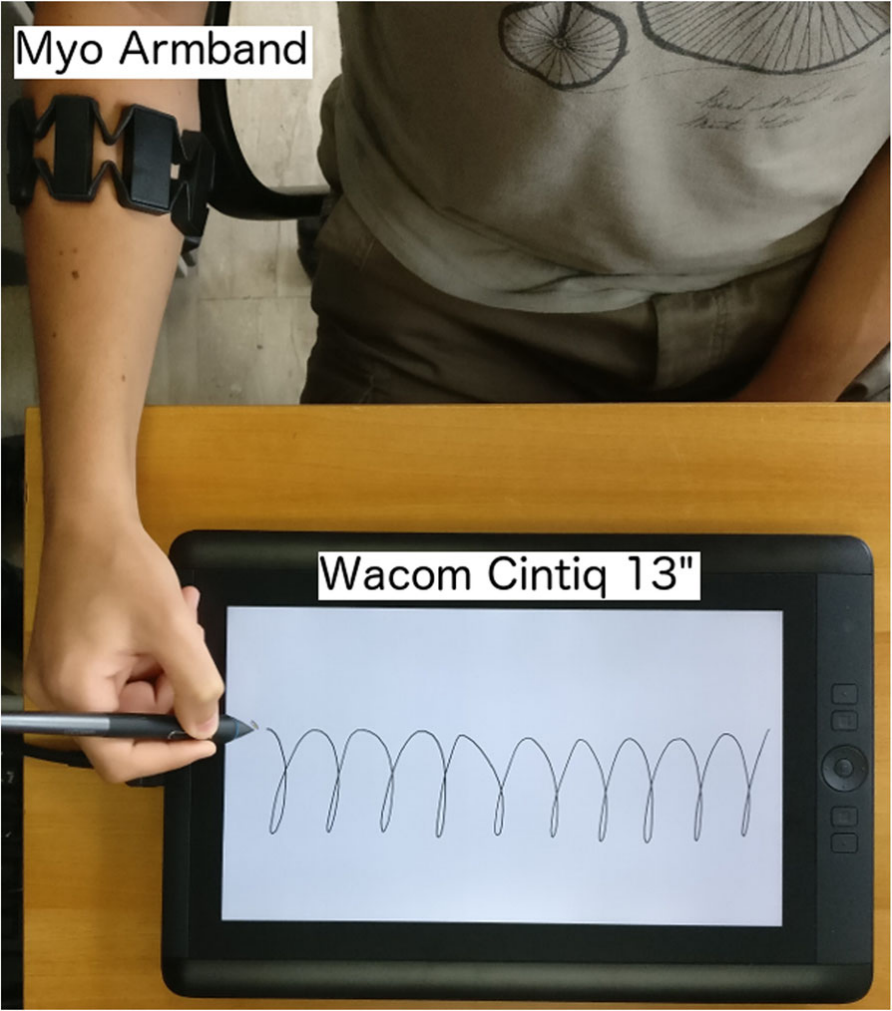
Example of the system set-up used for data acquisition

The data acquisition is based on the system set-up (Fig. 3) presented in a previous work [28]. The set-up is based on two sensors: the Myo™Gesture Control Armband [42] for sEMG signal acquisition and the graphics tablet WACOM Cintiq 13” HD [43] for acquiring handwriting signals and data. In detail, the Myo™Armband allowed us to acquire 8 different sEMG signals of the forearm, whereas the tablet permitted us to acquire the pen tip coordinates and pressure, and the tilt of the pen with respect to the writing surface.

### Experimental description

We used three writing patterns for the experiments leading to as many writing tasks. The patterns selected were as follows:

1. Pattern 1 – a five-turn spiral drawn in anticlockwise direction;
2. Pattern 2 – a sequence of 8 Latin letter ”l” with a size of 2.5cm;
3. Pattern 3 – a sequence of 8 Latin letter ”l” with a size of 5cm.

It is possible to observe that only two writing patterns (Patterns 2 and 3) were size-constrained, and for those, a visual marker has been provided as a size reference.

In the experiment, we asked each subject to perform four repetitions for each writing tasks: the first one was used to familiarise with the task and was discarded, the remaining three were recorded as data for the following processing.

Each handwriting tasks was interleaved with a rest period of three seconds, and the first pressure point on the tablet has been used as a trigger for the tasks begin.

Each subject generated 41 features for writing task 1 and to 43 features for writing task 2 and 3:

- Root Mean Square (RMS) of each sEMG signal (8 RMS features for each subject and for each task);
- Zero Crossing (ZC) of each sEMG signal (8 RMS features for each subject and for each task);

- mean and standard deviation for each subject and for each task of the following signals:
  - pen tip Cartesian velocity (two features);
  - X and Y velocity components of the pen tip (four features);
  - pen tip Cartesian acceleration (two features);
  - X and Y acceleration components of the pen tip (four features);
  - pen tip Cartesian jerk (two features);
  - X and Y jerk components of the pen tip (four features);
  - pen tip pressure (two features);
  - pen azimuth (two features);
  - pen altitude (two features);
- pattern specific features:
  - index of precision of the spiral drawing (one feature);
  - size related features for the constrained patterns (three features).

### Experimental data processing description

The objectives of the conducted experiments were mainly two:

1. the first was to separate PD patients from healthy subjects;
2. the second was to correctly classify mild and moderate Parkinson patients.

For each objective, the features extracted during the experiments were grouped according to the following scheme:

- creation of three different feature datasets:
  - dataset A including only the features extracted from writing pattern 1 (41 features);
  - dataset B including only the features extracted from writing pattern 2 (43 features);
  - dataset C including only the features extracted from writing pattern 3 (43 features);
- application of a feature selection algorithm to reduce the number of the features;
- creation of six different new feature datasets:
  - Case 1. Dataset including all the feature of set A;
  - Case 2. Dataset including all the feature of set B;
  - Case 3. Dataset including all the feature of set C
  - Case 4. Dataset including only the features obtained by the application of the feature selection algorithm on dataset A;
  - Case 5. Dataset including only the features obtained by the application of the feature selection algorithm on dataset B;
  - Case 6. Dataset including only the features obtained by the application of the feature selection algorithm on dataset C.

To assess both objectives of the experiments, an Artificial Neural Network (ANN) classifier featuring an optimised topology provided a Multi-Objective Genetic Algorithm (MOGA) was used.

**Fig. 4:**
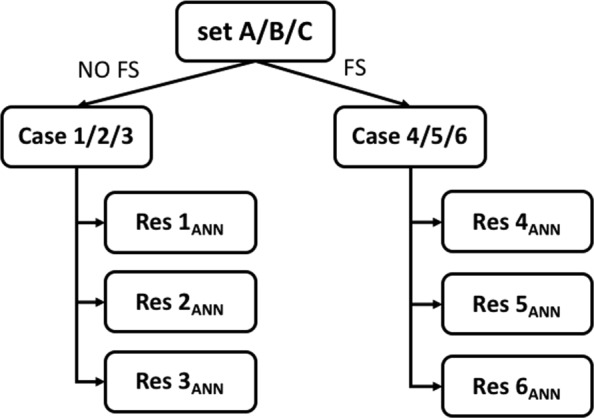
Scheme of the experiment. Features are grouped in three sets: A, B and C. The application of the Feature Selection (FS) algorithm leads to 6 cases

In detail, for each objective and each of the six cases, we estimated the performance indexes of the ANN optimal topology approach, reported in Eqs. 3, 4 and 5, in terms of percentage and standard deviation. Due to the dependence of the ANN performances from both net initialisation and permutation of training-validation datasets, we iterated the assessment over 250 repetitions of the net training procedure. Figure 4 depicts the scheme of the experiments.

### Results

**Fig. 5:**
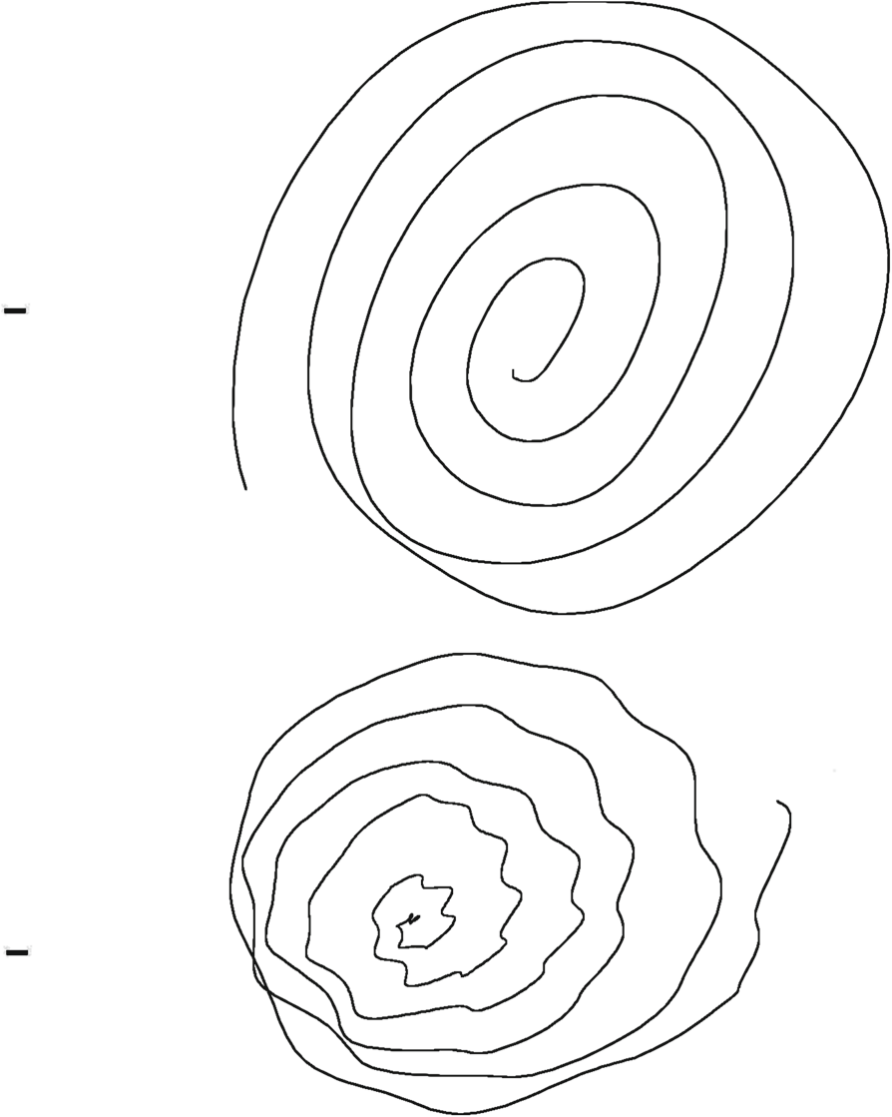
Example of one repetition of the spiral drawing task performed by a healthy subject (top) and a PD subject (bottom), respectively

Two samples of one repetition of the writing tasks performed respectively by healthy and PD subject are reported (not in real scale) in Figs. 5 (task no.1), 6 (task no.2) and 7 (task no.3).

**Fig. 6:**
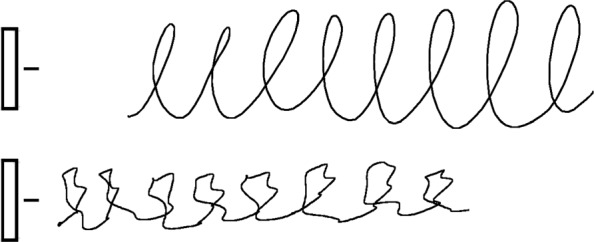
Example of one repetition of the letter-based task (sequence of eight "l" with size of 2.5cm) performed by a healthy subject (top) and a PD subject (bottom), respectively

**Fig. 7:**
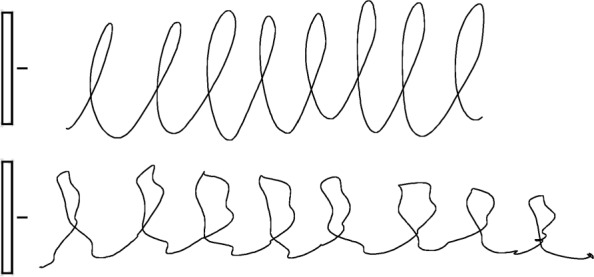
Example of one repetition of the letter-based task (sequence of eight "l" with size of 5cm) performed by a healthy subject (top) and a PD subject (bottom), respectively

Based on the two objectives the presentation and the discussion of the results obtained from the experiments have been subdivided. In particular, for each objective, we have reported the results obtained from both the feature selection and the classification for each of the six cases.

For each case the training procedure was iterated 250 times to assess the stability of the learning process; hence, the confusion matrices and the related results are presented in percentage with the standard deviation reported in brackets.

#### Objective 1 - separating PD patients and healthy subjects:

- *Feature Selection results*: The application of the feature selection algorithm previously reported, led to a significant reduction of the number of considered features for all three datasets of features extracted from the writing patterns. In particular:
- for dataset A including the 41 features extracted from writing pattern 1, the sEMG RMS value, three sEMG ZC values, the mean Cartesian velocity and the mean acceleration on X axes were the six selected features to be classified in Case 4;
- for dataset B including the 43 features extracted from writing pattern 2, the mean jerk on Y axes, three sEMG ZC values, the mean Cartesian acceleration and the mean velocity on X axes were the six selected features to be classified in Case 5;
- for dataset C including the 43 features extracted from writing pattern 3, two sEMG RMS values, a sEMG ZC value, the mean cartesian velocity, the altitude STD, the azimuth RMS and the mean velocity on X axes were the seven selected features to be classified in Case 6.
- *Classification results:* for each of the six different feature datasets, the MOGA algorithm is applied to provide the optimal ANN topology. The optimal topology results and the confusion matrices are reported in Table 2 and in Tables 3, 4, 5, 6, 7 and 8, respectively; the performances expressed in terms of accuracy, specificity and sensitivity have been summarised in Table 9.

**Table 2:** Objective 1: results of the application of the MOGA algorithm on each of the six different feature datasets

Case	Number of Features	Writing Pattern	ANN Topology		Accuracy
			Number of Neurons	Activation Function	
1	41	1	186/15/2	*logsig/logsig/softmax*	90.76%
2	43	2	44/10/2	*logsig/logsig/softmax*	92.98%
3	43	3	232/82/7/2	*logsig/logsig/logsig/softmax*	95.95%
4	6	1	222/25/2	*logsig/logsig/softmax*	93.78%
5	6	2	246/12/2	*logsig/logsig/softmax*	91.58%
6	7	3	45/114/21/2	*satlins/tansig/logsig/softmax*	96.85%

The output layer configuration was preliminarily fixed with two neurons and softmax as activation function

**Table 3:** Confusion matrix of Case 1 (Objective 1)

		True Condition	
		PD	Control
Predicted Condition	PD	*59.75%*	*5.35%*
	Control	*3.89%*	*31.02%*

**Table 4:** Confusion matrix of Case 2 (Objective 1)

		True Condition	
		PD	Control
Predicted Condition	PD	*60.36%*	*3.75%*
	Control	*3.89%*	*32.62%*

**Table 5:** Confusion matrix of Case 3 (Objective 1)

		True Condition	
		PD	Control
Predicted Condition	PD	*61.67%*	*2.09%*
	Control	*1.96%*	*34.27%*

**Table 6:** Confusion matrix of Case 4 (Objective 1)

		True Condition	
		PD	Control
Predicted Condition	PD	*61.40%*	*3.98%*
	Control	*2.24%*	*32.38%*

**Table 7:** Confusion matrix of Case 5 (Objective 1)

		True Condition	
		PD	Control
Predicted Condition	PD	*60.35%*	*5.13%*
	Control	*2.24%*	*31.24%*

**Table 8:** Confusion matrix of Case 6 (Objective 1)

		True Condition	
		PD	Control
Predicted Condition	PD	*62.11%*	*1.62%*
	Control	*1.53%*	*34.75%*

**Table 9:** Objective 1: performances of the application of the MOGA algorithm on each of the six different feature datasets

Case	Accuracy	Specificity	Sensitivity
1	0.9076 [0.0764]	0.8530 [0.1553]	0.9389 [0.0720]
2	0.9298 [0.0523]	0.8970 [0.1212]	0.9486 [0.0587]
3	0.9595 [0.0479]	0.9425 [0.0831]	0.9691 [0.0575]
4	0.9378 [0.0566]	0.8905 [0.1356]	0.9649 [0.0537]
5	0.9158 [0.0526]	0.8590 [0.1153]	0.9483 [0.0607]
6	0.9685 [0.0405]	0.9555 [0.0805]	0.9760 [0.0500]

Results are reported as man and standard deviation values over 250 iterations

#### Objective 2 - separating mild and moderate PD patients:

- *Feature Selection results:* the application of the feature selection algorithm previously reported, led to a significant reduction of the number of considered features for all three datasets of features extracted from writing patterns. In particular:
  - for dataset A including the 41 features extracted from writing pattern 1, two sEMG RMS values, two sEMG ZC values, the mean pressure and the mean altitude were the six selected features to be classified in Case 4;
  - for dataset B including the 43 features extracted from writing pattern 2, two sEMG RMS values, two sEMG ZC values and the mean Cartesian velocity were the five selected features to be classified in Case 5;
  - for dataset C including the 43 features extracted from writing pattern 3, two sEMG RMS values, a sEMG ZC value, the mean Cartesian velocity on X axes and the mean pressure were the five selected features to be classified in Case 6.
- *Classification results:* for each of the six different feature datasets, the MOGA algorithm is applied to provide the optimal ANN topology. The optimal topology results and the confusion matrices are reported in Table 10 and in Tables 11, 12, 13, 14, 15 and 16, respectively; the performances expressed in terms of accuracy, specificity and sensitivity have been summarised in Table 17.

**Table 10:** Objective 2: results of the application of the MOGA algorithm on each of the six different feature datasets

Case	Number of Features	Writing Pattern	ANN Topology		Accuracy
			Number of Neurons	Activation Function	
1	41	1	59/65/2/2	*logsig/logsig/logsig/softmax*	94.34%
2	43	2	138/18/1/2	*logsig/logsig/logsig/softmax*	87.26%
3	43	3	65/36/7/2	*logsig/logsig/logsig/softmax*	91.86%
4	6	1	123/2	*logsig/softmax*	96.00%
5	5	2	67/24/2	*logsig/logsig/softmax*	86.71%
6	5	3	17/2	*tansig/softmax*	91.66%

The output layer configuration was preliminarily fixed with two neurons and softmax as activation function

**Table 11:** Confusion matrix of Case 1 (Objective 2)

		True Condition	
		Moderate	Mild
Predicted Condition	Moderate	*39.51%*	*2.31%*
	Mild	*3.34%*	*54.83%*

**Table 12:** Confusion matrix of Case 2 (Objective 2)

		True Condition	
		Moderate	Mild
Predicted Condition	Moderate	*37.43%*	*7.31%*
	Mild	*5.43%*	*49.83%*

**Table 13:** Confusion matrix of Case 3 (Objective 2)

		True Condition	
		Moderate	Mild
Predicted Condition	Moderate	*39.51%*	*4.80%*
	Mild	*3.34%*	*52.34%*

**Table 14:** Confusion matrix of Case 4 (Objective 2)

		True Condition	
		Moderate	Mild
Predicted Condition	Moderate	*41.31%*	*2.46%*
	Mild	*1.54%*	*54.69%*

**Table 15:** Confusion matrix of Case 5 (Objective 2)

		True Condition	
		Moderate	Mild
Predicted Condition	Moderate	*36.51%*	*6.94%*
	Mild	*6.34%*	*50.20%*

**Table 16:** Confusion matrix of Case 6 (Objective 2)

		True Condition	
		Moderate	Mild
Predicted Condition	Moderate	*39.29%*	*4.77%*
	Mild	*3.57%*	*52.37%*

**Table 17:** Objective 2: performances of the application of the MOGA algorithm on each of the six different feature datasets

Case	Accuracy	Specificity	Sensitivity
1	0.9434 [0.0626]	0.9595 [0.0763]	0.9220 [0.1158]
2	0.8726 [0.0850]	0.8720 [0.1206]	0.8733 [0.1544]
3	0.9186 [0.0830]	0.9196 [0.1167]	0.9220 [0.1286]
4	0.9600 [0.0658]	0.9570 [0.0939]	0.9640 [0.0947]
5	0.8671 [0.0837]	0.8785 [0.1128]	0.8520 [0.1598]
6	0.9166 [0.0858]	0.9165 [0.1163]	0.9167 [0.1313]

Results are reported as mean and standard deviation values over 250 iterations

## Discussion

For the sake of clarity, we summarised the accuracy of all cases for both objectives in Table 18. As reported in the table, the proposed procedure leads to high accuracy performances; the results for both objectives present accuracy in the range 86<*x*<97, with a standard deviation lower than 0.09. The low value of the standard deviation allows us to assess the stability of the learning process of the optimal ANN. Similar observations can be stated for both objectives for the classification of the selected features. In detail:

- the classification between PD patients and healthy subjects (objective 1) achieves the best accuracy (96.85%) in *Case 6* (seven features selected from the dataset of 43 features extracted from writing pattern 3). The feature selection stated that three out of seven features were related to sEMG signals (RMS and ZC), whereas the others to pen tilt and velocity;

**Table 18:** Summary of the accuracy values obtained for each of the two objectives for each considered case

	Case	Objective	
		1	2
All Feature	1	90.76% (0.0764)	94.34% (0.0626)
	2	92.98% (0.0523)	87.26% (0.0850)
	3	95.95% (0.0479)	91.86% (0.0830)
Selected Feature	4	93.78% (0.0566)	96.00% (0.0658)
	5	91.58% (0.0526)	86.71% (0.0837)
	6	96.85% (0.0405)	91.66% (0.0858)

Standard deviation over 250 repetitions is reported in brackets
- the classification between mild and moderate PD patients (objective 2) achieves the best accuracy (96.00%) in *Case 4* (six features selected from the dataset of 41 features extracted from writing pattern 1). The feature selection stated that four out of six features were related to sEMG signals (RMS and ZC), whereas the others to pen tilt and velocity;

## Conclusions

In this work, we proposed an innovative methodology for computer-assisted handwriting analysis with the main goal of PD detection (healthy subjects vs. PD patients) and rating (mild vs moderate PD patients). The proposed approach is based on extracting different features from biometric signals collected during handwriting tasks and using such features to detect and rate PD. The developed decision support system (DSS) is based on an artificial neural network whose topology has been optimized with a MOGA.

The results showed that the proposed DSS is able to classify healthy subjects vs PD patients and mild vs moderate PD patients with a high classification accuracy (more than 90.0%). Furthermore, we proved that a limited set of representative feature selected by means of a classification decision tree technique, that uses the Gini’s diversity index, improves the overall accuracy (more than 96.0%).

Future works are needed to investigate the DSS performance with a larger cohort of subjects that includes severe PD patients too. This will allow us to classify PD patients by using more than two PD status classes and to monitor the progress of the disease over time. Furthermore, due to the time-consuming acquisition steps, it is desirable to reduce the required number of pattern tasks; this will be achieved through a proper writing pattern selection among the proposed ones.

## Data Availability

The datasets generated and analysed during the current study are not publicly available due to restrictions associated with anonymity of participants but are available from the corresponding author on reasonable request.

## Abbreviations

ANN: Artificial neural network
AUC: Area under the curve
FN: False negative
FP: False positive
MOGA: Multi-objective genetic algorithm
PD: Parkinson’s disease
RMS: Root mean square
ROC: Receiver operating characteristic
SEMG: surface electromyography
TN: True negative
TP: True positive
ZC: Zero crossing
